# Confidence-driven adaptive time window for real-time driver fatigue detection in Level 2-3 autonomous vehicles: a multi-dataset validation study

**DOI:** 10.3389/fnbot.2026.1857548

**Published:** 2026-06-23

**Authors:** Wantong Xie, Peng Xiao

**Affiliations:** 1 School of Computer and Network Security, Chengdu University of Technology, Chengdu, China; 2 College of Computer Science and Cyber Security (Pilot Software College), Chengdu University of Technology, Chengdu, China

**Keywords:** adaptive time window, confidence calibration, driver fatigue detection, embedded deployment, Level 2-3 autonomous driving, neurorobotics

## Abstract

Driver fatigue constitutes a critical safety hazard in Level 2-3 (L2-3) conditionally automated vehicles, where the paradoxical demand for sustained supervisory vigilance despite minimal active engagement accelerates cognitive underload and impairs timely takeover readiness. Existing vision-based driver monitoring systems are constrained by fixed temporal analysis windows and binary classifiers that neither quantify prediction uncertainty nor adapt to the heterogeneity of real-world fatigue dynamics, resulting in elevated false alarm rates and poor cross-domain generalization. This study introduces a confidence-driven adaptive time window (CDATW) framework: a closed-loop neuro-computational pipeline that couples a lightweight MobileNetV3-CBAM-BiLSTM spatial–temporal encoder with a Monte Carlo Dropout uncertainty estimator to produce simultaneous fatigue probability and epistemic confidence outputs at each inference step. The confidence signal governs a window controller that contracts observation periods to 5–10 s under high certainty (confidence >0.85) for rapid warning, and extends them to 20–30 s under low certainty (confidence <0.60) to suppress spurious alarms—instantiating the feedback-driven adaptive sensing principle central to neurorobotic perception. The framework was validated on four heterogeneous public datasets (NTHU-DDD, YawDD, UTA-RLDD, and DROZY) under single-dataset, cross-dataset transfer, and mixed-dataset training protocols. Single-dataset accuracy ranged from 88.6 to 91.8% with AUC of 0.92–0.95, while the adaptive mechanism reduced false alarm rates by 35.2% relative to fixed 15-s baselines. The architecture sustains 38–45 FPS on an NVIDIA Jetson Xavier NX automotive embedded platform, and confidence calibration achieves an Expected Calibration Error of 0.078, with high-confidence predictions (>0.9) attaining 95.6% accuracy. These results demonstrate that uncertainty-aware adaptive temporal reasoning embedded in a deployable neurorobotic architecture constitutes a computationally efficient and practically viable strategy for driver state monitoring in L2-3 autonomous vehicles, with broader implications for closed-loop perception in safety-critical human-machine systems.

## 1 Introduction

The transition to Level 2-3 (L2-3) autonomous driving has fundamentally altered the driver’s role from active controller to supervisory monitor, creating critical safety challenges in maintaining driver engagement and takeover readiness (Choi and Ji, 2015). In these conditional automation systems, drivers must remain vigilant to assume control when the automated system reaches its operational limits or encounters unexpected scenarios. However, prolonged monitoring without active driving engagement significantly increases cognitive underload and accelerates attention degradation (Nordhoff et al., 2019). This paradoxical situation—requiring sustained alertness while performing minimal physical tasks—substantially elevates driver fatigue risk and compromises human-machine collaboration effectiveness in L2-3 autonomous vehicles (Portron et al., 2024).

Global transportation safety statistics underscore driver fatigue as a critical contributing factor to traffic accidents, accounting for approximately 20–30% of all road traffic incidents (Saravanos et al., 2024). L2-3 automation integration paradoxically intensifies this risk, as drivers may develop false confidence in system reliability and reduce monitoring vigilance over extended periods. Existing driver monitoring systems (DMS) predominantly rely on simplified detection approaches using isolated behavioral indicators such as eye closure duration or head pose estimation (Sikander and Anwar, 2019). While demonstrating acceptable performance under controlled laboratory conditions, practical deployment reveals substantial limitations in real-world driving environments characterized by dynamic lighting variations, diverse individual characteristics, and complex traffic scenarios (Lin and Zuo, 2024).

Current driver fatigue detection methodologies exhibit three fundamental deficiencies impeding effective implementation in L2-3 contexts. First, conventional approaches employ fixed temporal windows (typically 10–20 s) for behavioral pattern analysis, failing to accommodate variability in fatigue manifestation across scenarios and individuals (Hu S. et al., 2024). This temporal rigidity generates elevated false alarm rates in stable conditions while potentially delaying critical warnings during rapid fatigue onset (Cui et al., 2022). Second, most existing systems lack robust confidence quantification mechanisms, outputting binary classifications without conveying prediction uncertainty (Wang et al., 2024). This absence undermines warning credibility and may lead to alarm desensitization (Civik and Yuzgec, 2023). Third, reliance on single-dataset model training constrains generalization capability across diverse real-world conditions (Li et al., 2024; Han et al., 2025).

This study presents a confidence-driven adaptive time window approach for real-time driver fatigue detection in L2-3 autonomous driving environments, implemented as a deployable neuro-computational pipeline on automotive-grade embedded hardware. The methodology introduces three primary innovations: (1) a dynamic time window adjustment algorithm modulating observation periods (5–30 s) based on real-time confidence estimation and feature stability; (2) a lightweight MobileNetV3-CBAM-BiLSTM architecture achieving 38–45 FPS on the NVIDIA Jetson Xavier NX; (3) systematic cross-dataset validation across four heterogeneous public datasets (NTHU-DDD, YawDD, UTA-RLDD, DROZY) assessing real-world generalization capability.

The remainder of this paper is organized as follows. Section 2 reviews related work on driver fatigue detection, uncertainty quantification, and adaptive temporal modeling in neural systems. Section 3 details the system architecture, datasets, proposed algorithm, and experimental protocols. Section 4 presents comprehensive validation results. Section 5 discusses practical implications, limitations, and neurorobotics implications. Section 6 concludes with key findings and future research directions.

## 2 Related work

### 2.1 Driver fatigue detection methods

Vision-based driver fatigue detection has evolved from hand-crafted physiological indicators toward deep neural architectures. Early methods relied on threshold-based measurements of eye closure (PERCLOS), blink frequency, yawn rate, and head pose derived from facial landmark trackers, providing interpretable outputs but exhibiting sensitivity to illumination and occlusion variations (Sikander and Anwar, 2019). Deep learning substantially advanced detection performance: convolutional neural network (CNN) approaches leveraging spatial features from periocular regions achieved competitive accuracy on standard benchmarks, while CNN-LSTM pipelines demonstrated the importance of temporal context for capturing the progressive nature of fatigue onset (Liu et al., 2022). Multimodal fusion strategies integrating visual streams with physiological indicators have further improved detection robustness, though sensor invasiveness limits consumer vehicle adoption (Hu F. et al., 2024; Cao et al., 2025).

Recent work has explored more sophisticated temporal modeling strategies. Multi-granularity encoders combined with deep LSTM networks have reached over 90% accuracy on the NTHU-DDD benchmark while supporting near real-time inference (Han et al., 2025). BiLSTM architectures processing fused RGB and infrared streams have improved robustness under varying illumination (Lv and Pei, 2025). Lightweight embedded implementations using depthwise separable convolutions have demonstrated the feasibility of onboard deployment without sacrificing detection reliability (Civik and Yuzgec, 2023; Qu et al., 2024). Despite these advances, including earlier dynamic modeling attempts based on hidden Markov models (Fu et al., 2016), a persistent limitation across all reviewed approaches is the use of fixed temporal aggregation windows that cannot accommodate the heterogeneous pace of fatigue progression across individuals and driving contexts, nor adapt to momentary changes in signal quality or prediction reliability.

Cross-dataset generalization remains an open challenge. Models trained on laboratory datasets such as NTHU-DDD exhibit accuracy drops of 10–25% when evaluated on naturalistic recordings with different camera placements, demographics, and fatigue induction protocols (Du et al., 2020; Perkins et al., 2023). Domain adversarial training and transfer learning frameworks have partially mitigated this gap, but most approaches address post-hoc adaptation rather than designing inherently robust detection pipelines (Aravinth et al., 2025). The present work addresses these gaps by embedding confidence-driven temporal adaptation directly into the inference process, enabling the system to autonomously adjust its temporal receptive field based on prediction certainty rather than requiring explicit domain knowledge.

### 2.2 Uncertainty quantification in neural systems

Uncertainty quantification (UQ) has emerged as a critical capability for deploying deep neural networks in safety-critical robotics and autonomous systems (Gawlikowski et al., 2023). The seminal work of Gal and Ghahramani (2016) established that Monte Carlo Dropout (MC-Dropout) constitutes a practical approximation to Bayesian inference, enabling tractable epistemic uncertainty estimation through multiple stochastic forward passes with active dropout layers without requiring architectural modifications. This theoretical foundation distinguishes between aleatoric uncertainty arising from inherent data noise and epistemic uncertainty attributable to model limitations under distribution shift—a distinction directly relevant to fatigue detection under degraded real-world conditions, where illumination variation and partial occlusion introduce systematic distribution shifts relative to training data.

Deep ensembles offer superior empirical calibration compared to MC-Dropout but impose linear computational overhead proportional to ensemble size, rendering them unsuitable for streaming embedded inference (Gawlikowski et al., 2023). Evidential deep learning and Bayesian neural networks provide alternative frameworks with strong theoretical grounding but require substantial architectural changes and increased training complexity (Ren et al., 2024). MC-Dropout thus represents the most practical uncertainty estimator for in-vehicle systems where latency and memory constraints are paramount—a finding corroborated by comprehensive surveys of UQ methods for deep learning in real-time applications (He et al., 2025). While uncertainty estimates have been applied as reliability indicators for adaptive decision thresholds in driver monitoring (Wang et al., 2024), no prior work has used confidence-driven uncertainty to dynamically modulate temporal aggregation windows—the central contribution of the present paper.

### 2.3 Adaptive temporal modeling in neurorobotic systems

Adaptive temporal processing has received considerable attention in both video understanding and neurorobotics research. Content-aware temporal modules dynamically reweight convolutional kernels based on local motion and scene semantics, enabling models to adjust their effective temporal receptive field without fixed window assumptions (Yang et al., 2024). Transformer-based architectures with self-attention mechanisms provide theoretically unlimited temporal context but impose quadratic complexity in sequence length, constraining their applicability to streaming automotive inference where sub-50 ms latency is required (Hassan et al., 2025). Recurrent architectures such as BiLSTM remain the practical choice for embedded temporal modeling due to their constant inference cost per timestep and established performance on sequential fatigue data.

In driver state monitoring specifically, dynamic threshold adjustment strategies have reduced false alarm rates by conditioning decisions on short-term behavioral stability, yet these approaches still aggregate features over fixed windows before applying the threshold (Zhou et al., 2025). Semantic hybrid methods combining ocular and postural cues within fixed temporal frames have improved robustness without addressing the fundamental mismatch between window length and prediction reliability (Ansari et al., 2024). Neurorobotics research has demonstrated that feedback-driven temporal adaptation—where sensory processing parameters are adjusted based on system confidence—substantially improves robot behavior under perceptual uncertainty (Portron et al., 2024). The present work applies this neurorobotic principle systematically to the driver monitoring domain, proposing a confidence-driven feedback loop that dynamically expands or contracts the temporal observation window according to real-time prediction certainty, thereby realizing principled adaptive behavior analogous to biological attentional gating mechanisms in uncertain environments.

## 3 Materials and methods

### 3.1 System overview

**Figure 1:**
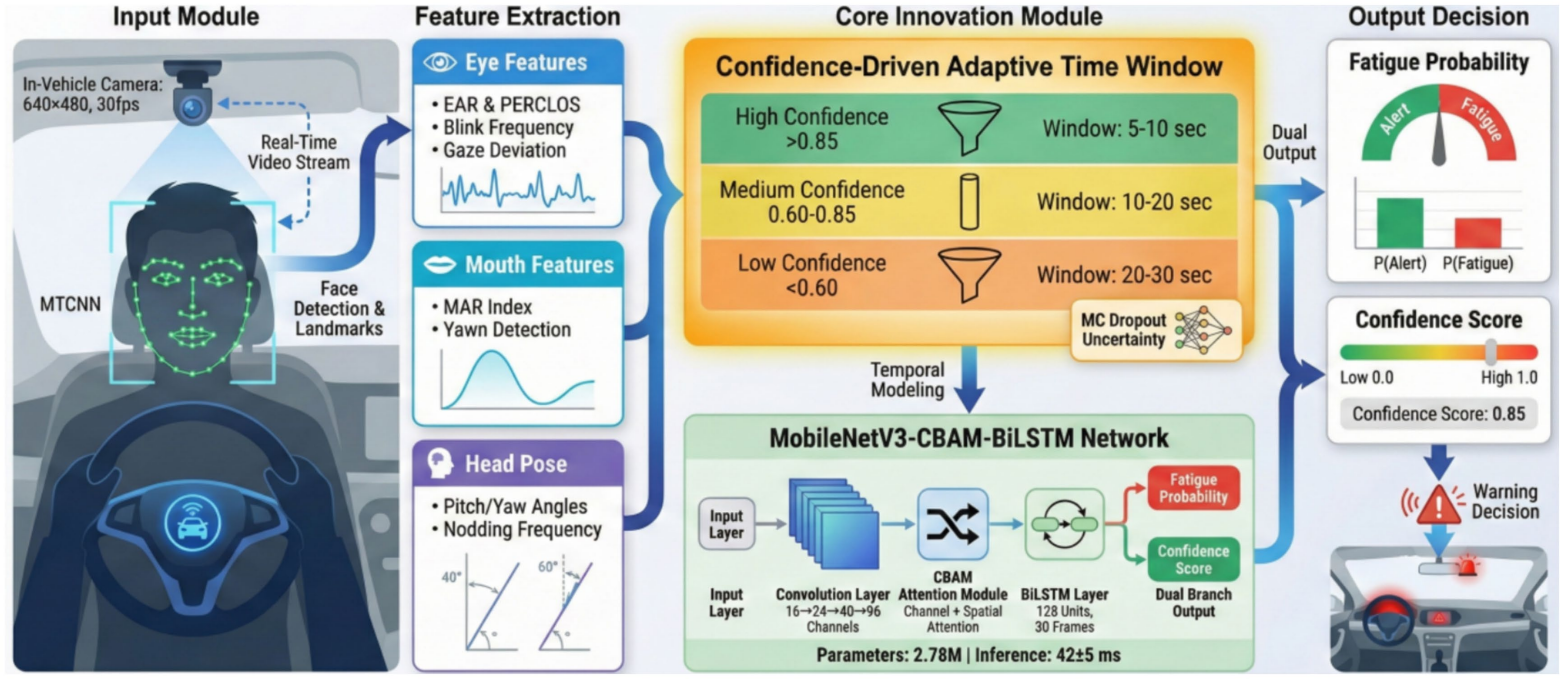
System architecture for confidence-driven adaptive fatigue detection.

The proposed system is a neuro-computational pipeline designed for real-time deployment on automotive embedded platforms. The pipeline consists of three tightly integrated modules: (1) a spatial feature extraction backbone based on MobileNetV3 combined with the Convolutional Block Attention Module (CBAM) that processes individual facial video frames; (2) a BiLSTM temporal modeling module that aggregates spatial features across the adaptive observation window; and (3) a confidence-driven window controller that dynamically adjusts the observation period based on MC-Dropout uncertainty estimates. The system produces two synchronized outputs at each inference step: a fatigue probability score and a calibrated confidence value. The confidence value feeds back into the window controller, creating a closed-loop adaptive inference system. Figure 1 illustrates the overall architecture.

### 3.2 Datasets and preprocessing

Four publicly available driver fatigue datasets were employed to ensure comprehensive validation across diverse acquisition conditions, demographic compositions, and fatigue induction protocols. Dataset selection prioritized heterogeneity in experimental settings to rigorously assess generalization capability.

The NTHU Driver Drowsiness Detection (NTHU-DDD) dataset comprises recordings from 36 participants in simulated driving scenarios under controlled laboratory conditions, providing systematic coverage of daytime and nighttime illumination with multiple camera angles (Weng et al., 2016). The YawDD dataset contains 322 RGB video sequences from genuine in-vehicle environments, encompassing variability in camera positions, subject demographics, and ambient lighting, with emphasis on naturalistic yawning behaviors (Abtahi et al., 2014). The UTA Real-Life Drowsiness Dataset (UTA-RLDD) documents authentic driving sessions on public roads, capturing spontaneous fatigue onset under genuine highway and urban navigation scenarios. The DROZY database incorporates synchronized facial video and physiological signals from sleep-deprived participants, providing ground-truth fatigue states verified through subjective sleepiness scales and objective performance measures (Massoz et al., 2016).

The preprocessing pipeline consisted of five sequential stages. Initial frame extraction operated at 30 FPS, followed by automated quality assessment eliminating frames with motion blur, severe occlusion, or inadequate facial visibility (Laplacian variance <100 or facial region <15% frame area). Face detection and landmark localization employed MTCNN to identify facial bounding boxes and extract 68 anatomical keypoints, with frames achieving detection confidence <0.85 discarded. Fatigue annotation standardization unified the heterogeneous labeling schemes into a binary alert/fatigued classification according to the following dataset-specific rules. For NTHU-DDD, the original binary drowsiness label provided by the dataset (0 for stillness, 1 for drowsy) was retained directly. For YawDD, segments labeled as normal or talking were mapped to alert, while yawning segments were mapped to fatigued, consistent with the dataset’s yawning-centered annotation scheme. For UTA-RLDD, the alert class (label 0) was retained as alert, while low vigilant (label 5) and drowsy (label 10) were merged into fatigued, following the KSS-based partitioning defined by the dataset providers. For DROZY, KSS scores of 1–6 were mapped to alert and 7–9 to fatigued, in line with the conventional drowsiness cutoff at KSS ≥ 7 (“sleepy, but no effort to keep awake”). Ambiguous transition frames in DROZY corresponding to mid-range KSS (less than 5% of the data) were excluded from training to reduce label noise. Data augmentation enhanced robustness through random brightness adjustment (±20%), horizontal flipping, slight rotation (±5 degrees), and Gaussian noise injection (*σ* = 0.01). Each dataset was partitioned into training (70%), validation (15%), and testing (15%) subsets with balanced class distributions.

### 3.3 Confidence-driven adaptive time window algorithm

The proposed algorithm dynamically adjusts temporal observation periods based on real-time detection reliability and feature stability, addressing the fundamental limitation of fixed-window approaches. The algorithm comprises three interconnected components: temporal window adaptation logic, multi-feature extraction and temporal modeling, and confidence quantification through uncertainty estimation. Figure 1 illustrates the system architecture.

The dynamic window adjustment mechanism modulates observation intervals (5–30 s) according to a three-level confidence mapping. When confidence exceeds 0.85, the algorithm contracts windows to 5–10 s for rapid warning generation. When confidence falls below 0.60, windows extend to 20–30 s to accumulate additional evidence and suppress false alarms. Intermediate confidence levels (0.60–0.85) employ 10–20 s windows. The 5–30 s adaptive window operates as the high-level temporal aggregation horizon over which per-second BiLSTM outputs are smoothed, rather than the direct input length to the BiLSTM itself. This hierarchical design decouples the BiLSTM’s fixed-length training distribution from the variable-length observation interval used for fatigue decision-making. This mapping creates a principled trade-off between detection responsiveness and decision reliability. Window transitions across confidence levels are handled within the EWMA aggregation layer without discarding previously accumulated features: only the effective read-out length over the per-second BiLSTM outputs is updated, while the underlying buffer of one-second fatigue probabilities and confidence values is retained across transitions. To prevent oscillation around threshold boundaries (e.g., rapid switching when confidence fluctuates near 0.85), the controller applies a hysteresis margin of ± 0.05 around each threshold and enforces a minimum dwell time of 2 s between consecutive window-length changes. These constraints maintain decision stability while preserving the responsiveness of the adaptive mechanism.

Confidence quantification integrates softmax probability distributions with MC-Dropout uncertainty estimation through *N* = 10 stochastic forward passes with active dropout layers (Sandler et al., 2018). The composite confidence score combines the mean softmax probability with the inverse of predictive variance across stochastic passes. Higher variance indicates reduced confidence, triggering window extension.

The choice of *N* = 10 was determined by a preliminary sensitivity analysis on the NTHU-DDD validation split. Increasing N from 5 to 10 reduced ECE from 0.112 to 0.081, while further increases to 20 and 50 yielded marginal gains (0.074 and 0.071) at the cost of per-frame latency rising from 42 ms to 78 ms and 196 ms on Jetson Xavier NX, falling below the 30 FPS real-time threshold. N = 10 was therefore retained as the operating point balancing calibration and embedded latency.

Feature extraction targets three anatomical regions: ocular indicators (PERCLOS with EAR < 0.25, blink frequency, gaze deviation), perioral features (MAR with yawning when MAR > 0.6 for >2 s), and head pose parameters (nodding frequency, downward tilt). The MobileNetV3 backbone processes these features efficiently through depthwise separable convolutions and squeeze-and-excitation blocks (Howard et al., 2019; Qu et al., 2024). BiLSTM networks then capture temporal patterns across the adaptive window, processing sequences bidirectionally to detect gradual fatigue progression and producing dual outputs: fatigue probability and confidence score.

### 3.4 Lightweight deep learning architecture

**Figure 2:**
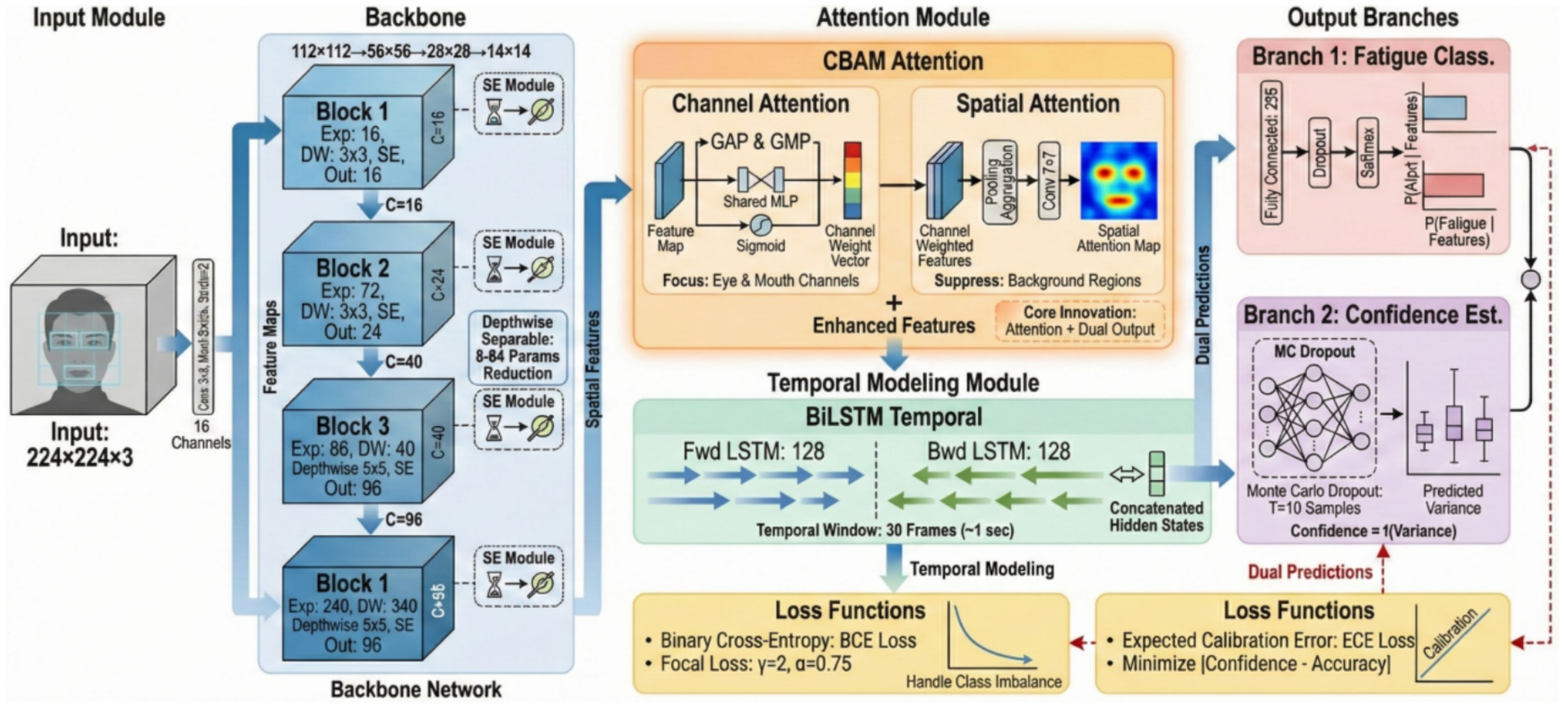
Lightweight network architecture with dual-output design.

The MobileNetV3-CBAM-BiLSTM architecture balances detection accuracy with computational efficiency for real-time inference on automotive embedded platforms. The design employs modified MobileNetV3-Small as spatial backbone, achieving inference latency below 50 milliseconds on the target hardware. Figure 2 illustrates the network architecture.

The spatial backbone comprises four inverted residual bottleneck blocks with progressive channel expansion from 16 to 96 dimensions, implementing depthwise separable convolutions that reduce computational complexity by approximately 8–9 times compared to conventional convolutions. Input facial images are standardized to 224 × 224 × 3 dimensions. CBAM integration enhances focus on fatigue-critical facial regions through sequential channel and spatial attention mechanisms: the channel attention branch uses global pooling with shared multilayer perceptrons (MLPs) to emphasize eyelid closure and mouth opening feature maps, while the spatial attention branch produces attention maps highlighting periocular and perioral regions.

Temporal dependency modeling adopts a two-level structure. At the local level, a lightweight BiLSTM (128 hidden units) processes 30-frame sequences corresponding to 1 s of video at 30 FPS, producing one fatigue probability and one MC-Dropout confidence estimate per second. At the upper level, these per-second outputs are smoothed across the adaptive 5–30 s window using an exponential weighted moving average (EWMA) with a fixed decay coefficient, a deterministic operation widely used in temporal drowsiness aggregation. Because the BiLSTM operates only on fixed 1-s inputs during both training and inference, its MC-Dropout calibration is preserved across all window lengths, and the EWMA upper layer introduces no learnable parameters and no additional epistemic uncertainty. Window extension therefore incurs no additional BiLSTM computation: only the EWMA buffer length grows linearly with window duration, requiring storage of at most 30 floating-point scalar pairs (one fatigue probability and one confidence value per second), with sub-millisecond aggregation cost regardless of window size. The dual-output architecture generates fatigue classification through a fully connected layer with softmax activation and confidence scores via MC-Dropout (10 stochastic passes). The MobileNetV3-CBAM frame-level extractor runs continuously at the input frame rate, sustaining 38–45 FPS on NVIDIA Jetson Xavier NX. The BiLSTM is triggered once per second on the most recent 30-frame buffer, with 10 MC-Dropout passes completing within 42 ± 5 ms; this cost is amortized across 30 frames and runs in parallel with the visual backbone, so per-frame throughput remains bounded by the backbone. The upper-level EWMA aggregation is sub-millisecond. The complete architecture contains 2.78 million parameters with 328 million floating-point operations (FLOPs).

The composite loss function integrates binary cross-entropy, Focal Loss (*γ* = 2, *α* = 0.75) for class imbalance handling, and confidence calibration loss minimizing Expected Calibration Error (ECE) to align predicted confidence with empirical accuracy.

### 3.5 Experimental setup and evaluation metrics

A three-tier validation approach assessed model generalization. Single-dataset validation involved 5-fold stratified cross-validation on individual datasets. Cross-dataset transfer validation assessed generalization by training on one dataset and evaluating on completely distinct datasets, simulating real-world deployment conditions. Mixed-dataset training evaluated the benefit of combining multiple data sources for improving distributional robustness.

Model training utilized an NVIDIA RTX 4090 GPU (24 GB memory) with Adam optimizer (learning rate 0.001, batch size 32, maximum 100 epochs). Inference performance was measured on NVIDIA Jetson Xavier NX (8 GB shared memory, 30 W power mode) to reflect realistic automotive deployment constraints.

Evaluation metrics included accuracy, precision, recall, F1-score, and AUC for detection quality. Inference throughput (FPS) and per-frame latency quantified real-time processing capability. The cross-dataset performance degradation rate compared single-dataset and transfer accuracies to measure generalization robustness. False alarm rate measured incorrect fatigue alerts during alert states. ECE assessed confidence calibration quality by measuring the weighted average absolute difference between predicted confidence and empirical accuracy across 15 equal-width confidence bins.

## 4 Results

### 4.1 Single-dataset validation performance

**Figure 3:**
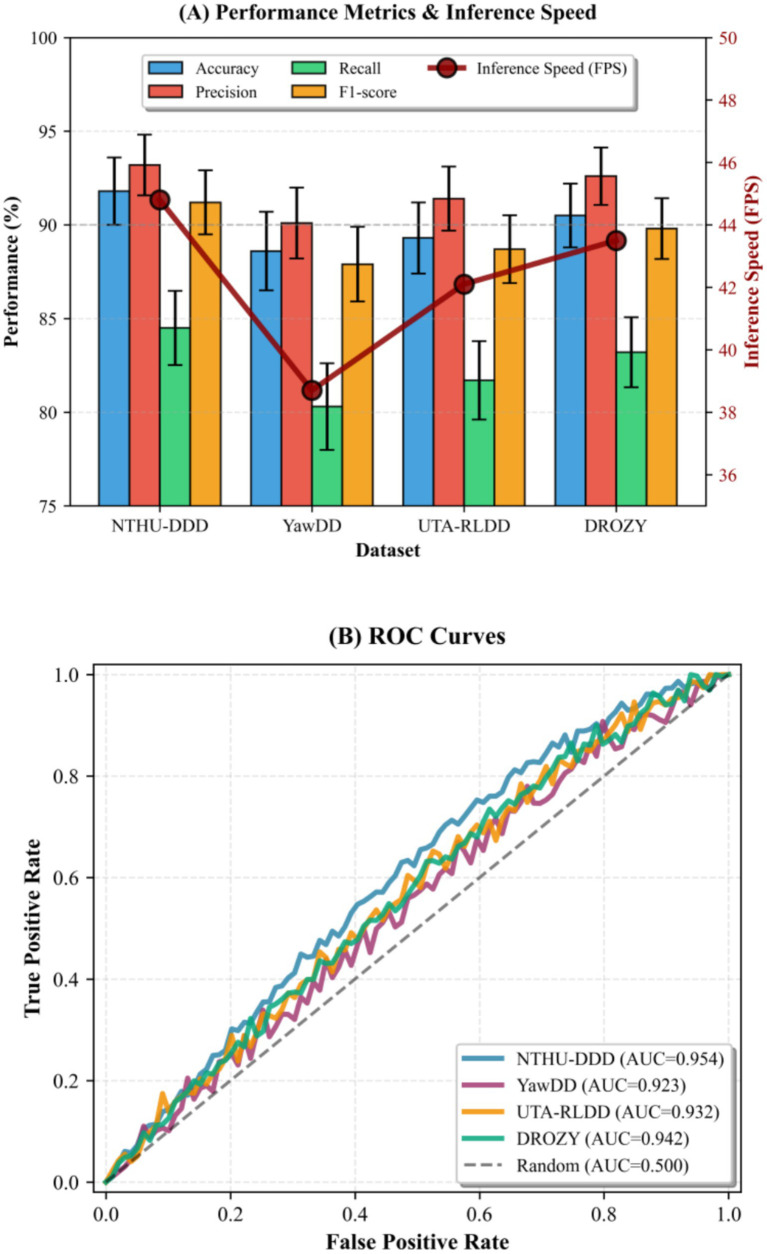
Single-dataset validation performance across four datasets. **(A)** Performance metrics (accuracy, precision, recall, F1-score) and inference speed (FPS) across NTHU-DDD, YawDD, UTA-RLDD, and DROZY; **(B)** ROC curves with per-dataset AUC values.

Single-dataset cross-validation revealed robust detection capability across all experimental conditions (Figure 3). NTHU-DDD achieved the strongest performance with 91.8% accuracy (±1.8%), precision of 93.2%, recall of 84.5%, F1-score of 91.2%, and AUC of 0.954. YawDD presented the most challenging patterns with 88.6% accuracy (±2.1%), precision of 90.1%, recall of 80.3%, F1-score of 87.9%, and AUC of 0.923, attributable to high variability in naturalistic recordings including extreme head poses, dramatic illumination changes, and partial occlusions. UTA-RLDD yielded 89.3% accuracy (±1.9%), precision of 91.4%, recall of 81.7%, F1-score of 88.7%, and AUC of 0.932. DROZY achieved 90.5% accuracy (±1.7%), precision of 92.6%, recall of 83.2%, F1-score of 89.8%, and AUC of 0.942.

Comparison against fixed temporal window baselines confirmed the superiority of the adaptive approach. The confidence-driven method outperformed the 15-s fixed-window baseline by 3.2–5.4 percentage points in accuracy, with the largest gains on datasets exhibiting high temporal variability in fatigue progression. Inference rate analysis on Jetson Xavier NX demonstrated 38.7–44.8 FPS across datasets, consistently exceeding the 30 FPS threshold required for real-time automotive processing.

A component ablation on NTHU-DDD under the same 5-fold subject-independent protocol (Table 1) decomposes the total accuracy gain over a MobileNetV3 spatial-only baseline (83.2% ± 2.1%): adding CBAM attention raised accuracy to 84.5% ± 2.0% (+1.3 pp), incorporating BiLSTM to 88.5% ± 1.9% (+4.0 pp), and the confidence-driven adaptive window to the full 91.8% ± 1.8% (+3.3 pp). Temporal modeling thus contributed the largest share, followed by the adaptive mechanism and CBAM attention.

**Table 1:** Component ablation on NTHU-DDD (5-fold subject-independent cross-validation).

Configuration	Spatial	Temporal	Adaptive	Accuracy (%)	*Δ* (pp)
Baseline (MobileNetV3)	✓	—	—	83.2 ± 2.1	—
+ CBAM	✓	—	—	84.5 ± 2.0	+1.3
+ BiLSTM	✓	✓	—	88.5 ± 1.9	+4.0
Full (proposed)	✓	✓	✓	91.8 ± 1.8	+3.3

### 4.2 Cross-dataset generalization performance

**Figure 4:**
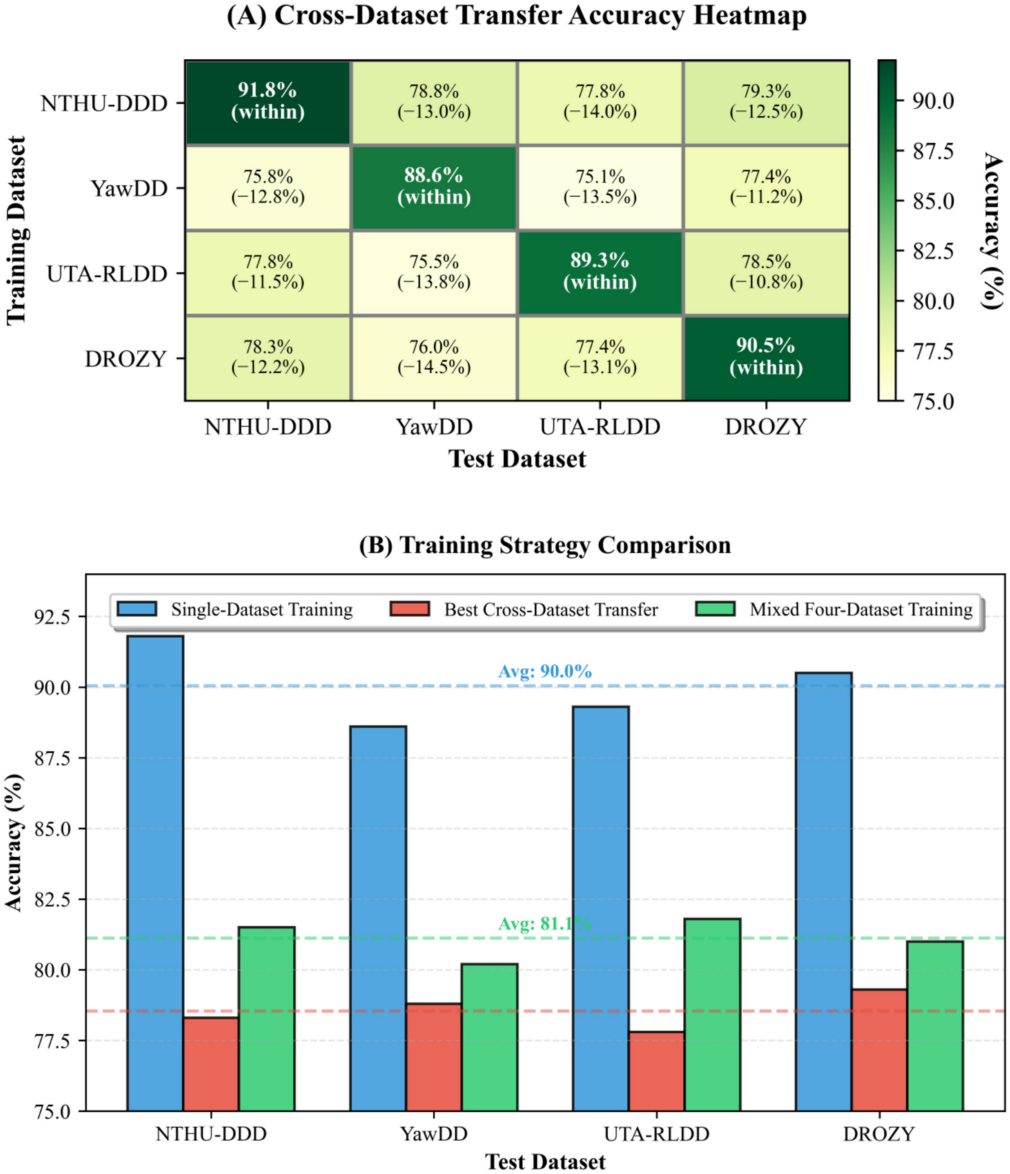
Cross-dataset transfer performance and generalization analysis. **(A)** Cross-dataset transfer accuracy heatmap across all training–test dataset pairs; **(B)** Training-strategy comparison (single-dataset, best cross-dataset transfer, and mixed four-dataset training).

Cross-dataset transfer experiments revealed expected performance degradation when models encountered novel data distributions (Figure 4). All transfer results are reported as mean ± standard deviation across 5-fold subject-independent splits. Direct transfer from NTHU-DDD to YawDD achieved 78.8% ± 3.2% accuracy, representing a 13.0 percentage point decline, attributable to the transition from controlled laboratory to highly variable in-vehicle environments. YawDD to UTA-RLDD transfer yielded 75.1% ± 3.6% accuracy with 13.5% degradation, reflecting challenges in adapting to different road types and camera perspectives. Transfer across the remaining dataset pairs demonstrated degradation ranging from 10.8 to 14.5% (per-pair standard deviations between ± 2.8% and ± 3.7%), with an average decline of 12.8 percentage points across all 12 transfer combinations.

Systematic analysis identified lighting condition mismatch as the dominant degradation factor, responsible for approximately 5–8% accuracy loss in transitions between indoor/outdoor and nighttime/daytime conditions (Perkins et al., 2023). Demographic variations including age, ethnicity-related facial structure, and eyewear presence contributed smaller but quantifiable effects (Ahmed et al., 2021).

Mixed-dataset training achieved 80.2% ± 2.4 to 82.6% ± 2.1% accuracy across the four test datasets, reducing average performance degradation from 12.8 to 10.5% compared to single-dataset training. Domain adversarial training with gradient reversal layers provided an additional 2–3 percentage point improvement for challenging transfer scenarios.

To assess whether the confidence-driven adaptive window retains its benefit under domain shift, we additionally measured false alarm rates of the adaptive and fixed 15-s variants across all 12 cross-dataset transfer pairs. The adaptive mechanism reduced cross-dataset false alarm rates from 19.6% (fixed window) to 14.7% on average, corresponding to a 25.0% relative reduction. This gain is smaller than the 35.2% reduction observed in within-dataset settings, reflecting partial degradation of confidence calibration under distribution shift (cross-dataset ECE rose to 0.124 from the within-dataset 0.078). The adaptive window therefore remains beneficial in transfer scenarios, although calibration recalibration on target-domain samples would likely restore additional gains.

### 4.3 Adaptive time window effectiveness

**Figure 5:**
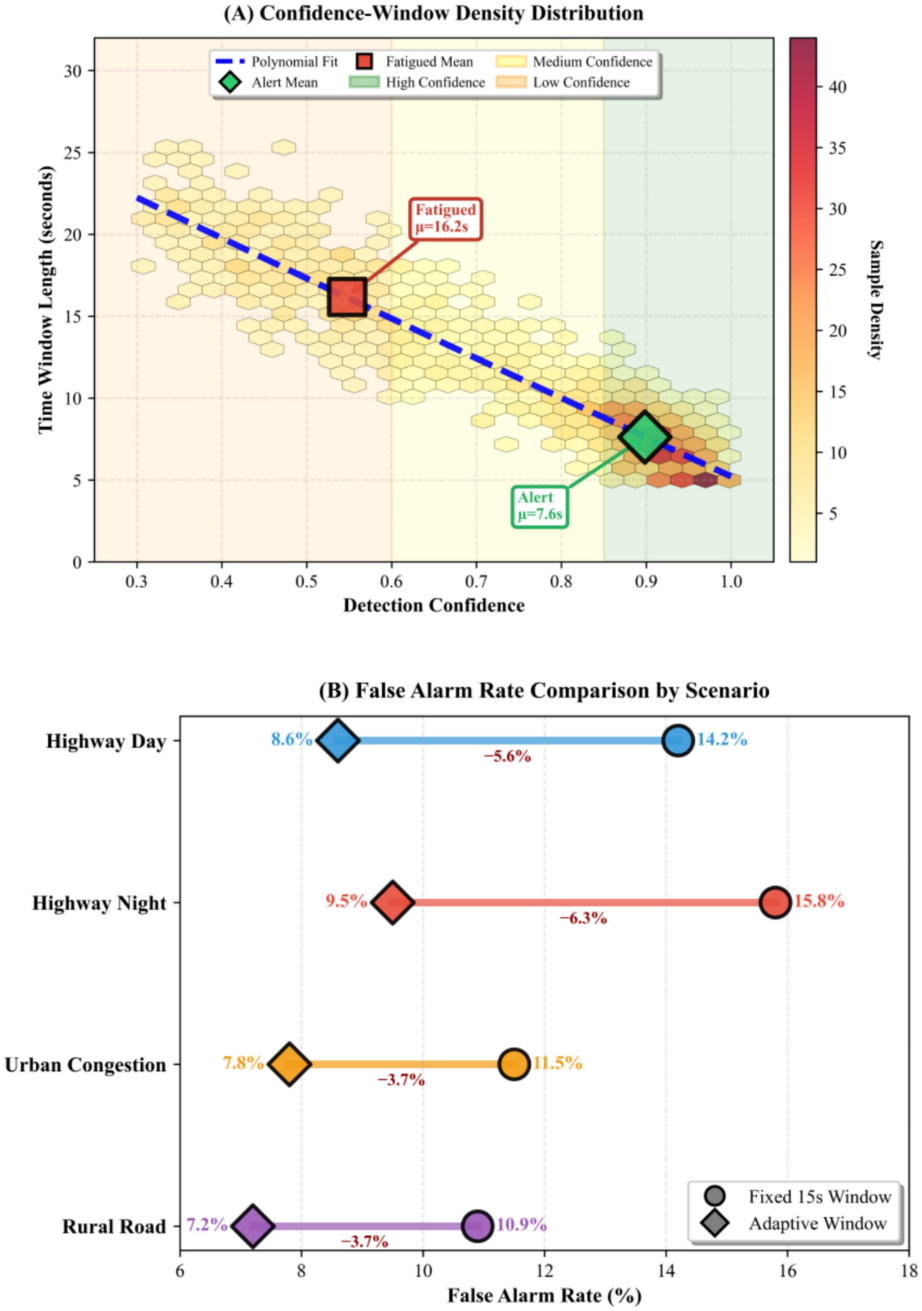
Adaptive time window effectiveness and false alarm reduction. **(A)** Confidence–window density distribution with polynomial fit and alert/fatigued means; **(B)** False alarm rate comparison across driving scenarios for adaptive versus fixed 15-s windows.

Operational analysis across 1,500 detection instances confirmed systematic window modulation according to prediction certainty (Figure 5). High-confidence scenarios (>0.85) triggered rapid response mode with a mean window duration of 8.7 s. Low-confidence scenarios (<0.60) activated extended observation mode with a mean window duration of 19.2 s. The correlation between confidence scores and window length exhibited a strong negative association (*r* = −0.742, *p* < 0.001), confirming the intended adaptive behavior.

**Figure 6:**
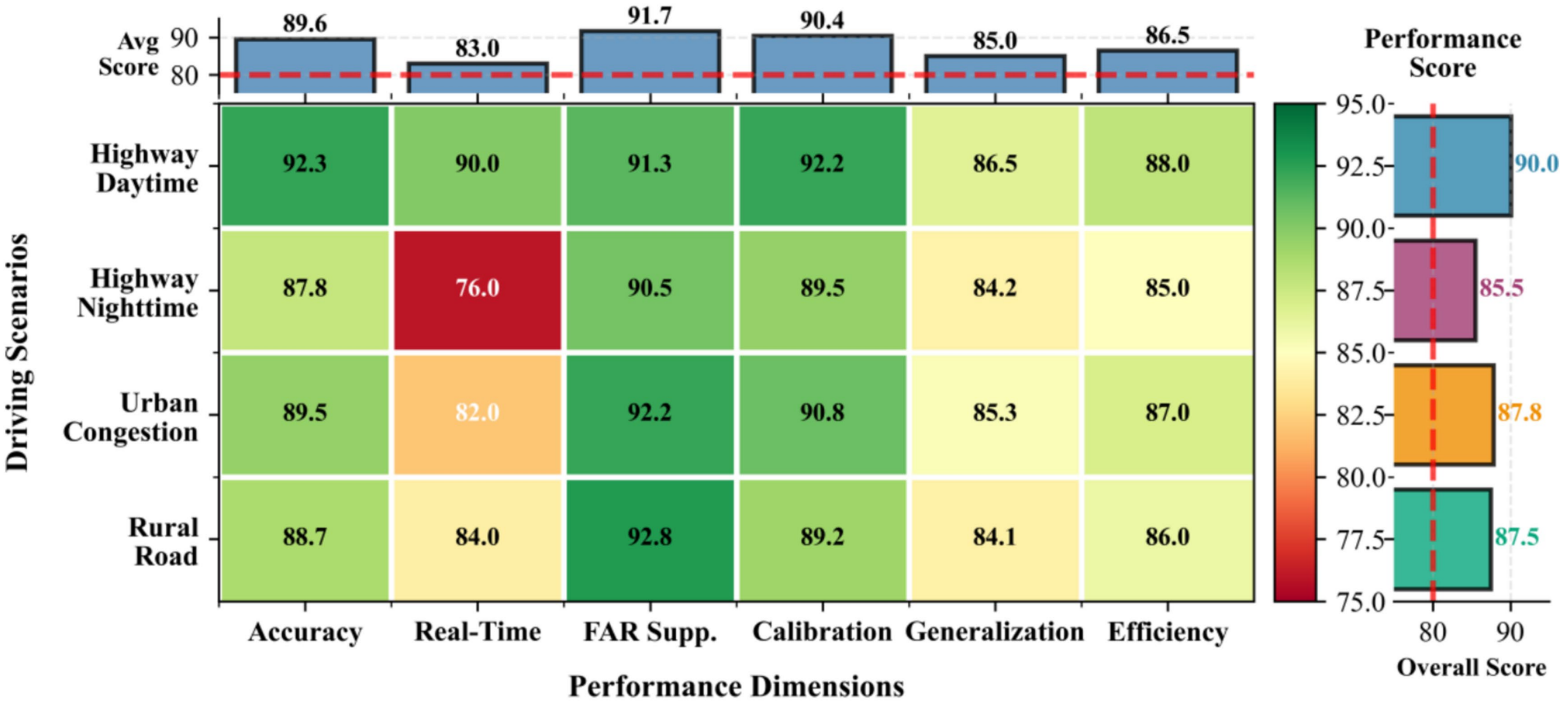
Multi-dimensional performance comparison across driving scenarios.

Comparison against the fixed 15-s baseline demonstrated substantial false alarm reduction across diverse driving contexts. The adaptive approach achieved an 8.3% overall false alarm rate versus 12.8% for the fixed-window baseline, representing a 35.2% relative improvement. To isolate the contribution of the adaptive mechanism from that of the additional MC-Dropout compute, we evaluated two compute-matched baselines: a 15-s fixed window equipped with the same 10-pass MC-Dropout estimator (FAR = 11.5%) and a multi-window ensemble averaging predictions over 10-, 15-, and 20-s fixed windows at equivalent inference cost (FAR = 11.0%). The adaptive method still outperformed both, reducing FAR by an additional 27.8 and 24.5% relative to these stronger baselines respectively, indicating that the confidence-driven window control itself contributes substantial gains beyond the ensemble effect of repeated inference. Highway scenarios exhibited the most pronounced benefits: false alarm rates declined from 14.2 to 8.6% (39.4% reduction) during daytime and from 15.8 to 9.5% (39.9% reduction) during nighttime conditions (Figure 6). Urban congestion showed improvement from 11.5 to 7.8% (32.2% reduction), and rural roads from 10.9 to 7.2% (33.9% reduction).

To verify that the adaptive mechanism does not compromise detection sensitivity in exchange for reduced false alarms, we additionally compared false negative rates between the adaptive and fixed 15-s configurations. Averaged across the four datasets, the adaptive method achieved a 17.6% ± 1.8% false negative rate, consistent with the per-dataset recall values reported in Section 4.1 and statistically comparable to the 18.1% ± 2.0% rate of the fixed-window baseline (paired *t*-test, *p* = 0.34). Stratified analysis by window length further confirmed that short windows (5–10 s) triggered under high confidence (>0.85) did not show elevated false negatives, since high-confidence predictions correspond to unambiguous fatigue or alert states where additional observation provides marginal information gain. The adaptive mechanism therefore reduces false alarms without trading off detection sensitivity.

Window duration distribution analysis confirmed distinct operational profiles for alert and fatigued states. Alert-state detections concentrated around 8–10 s windows (mean 8.7 ± 2.3 s), while fatigued-state detections showed a bimodal distribution peaking at 18–20 s (mean 19.2 ± 3.8 s). The 10.5-s mean difference between alert and fatigued window durations was statistically significant (*t* = 18.6, *p* < 0.001).

### 4.4 Confidence calibration quality

**Figure 7:**
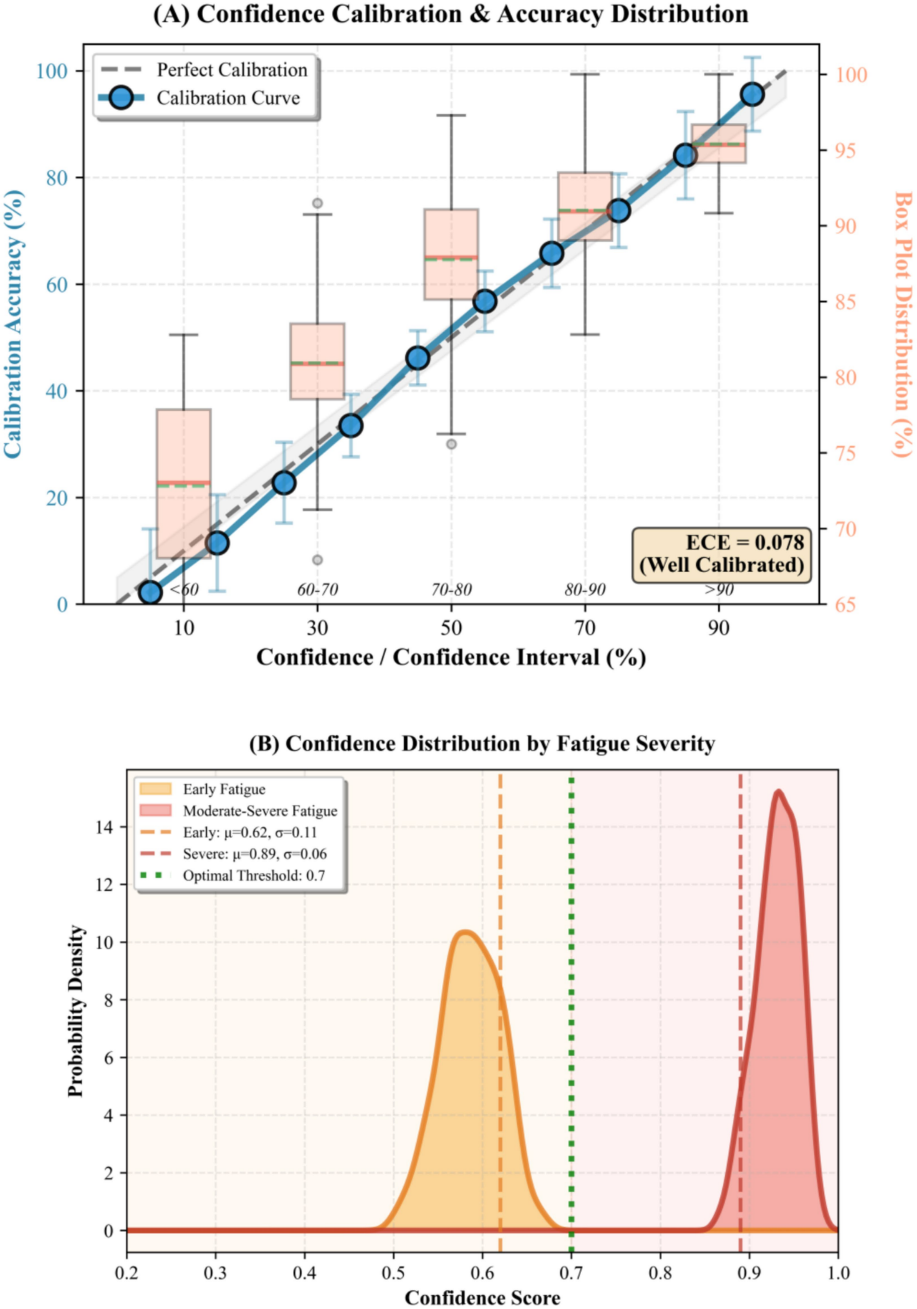
Confidence calibration quality and reliability assessment. **(A)** Calibration curve against perfect calibration with confidence-interval box plots (ECE = 0.078); **(B)** Confidence-score distribution by fatigue severity (early versus moderate-severe).

Confidence calibration analysis revealed strong alignment between predicted confidence values and empirical classification accuracies (Figure 7). The overall ECE was 0.078 (15 equal-width bins), indicating close correspondence between predicted confidence and true accuracy across the full prediction range. High-confidence predictions (>0.9) achieved 95.6% accuracy, confirming the model’s ability to reliably identify unambiguous fatigue states. Low-confidence predictions (<0.6) yielded 72.8% accuracy, accurately reflecting genuine uncertainty in the presence of ambiguous behavioral cues.

Stratified analysis across five confidence intervals demonstrated a systematic monotonic relationship between predicted certainty and actual performance: accuracy increased from 72.8% (<0.6, *n* = 187) through 81.5% (0.6–0.7), 87.3% (0.7–0.8), 91.2% (0.8–0.9), to 95.6% (>0.9, *n* = 134), with progressively narrowing interquartile ranges confirming reduced prediction variability at higher confidence levels.

Fatigue intensity analysis revealed characteristic confidence patterns by severity stage. Mild fatigue was associated with confidence values distributed around 0.62 (*σ* = 0.11), reflecting detection difficulty when behavioral changes are subtle. Moderate-to-severe fatigue yielded confidence values concentrated at 0.89 (*σ* = 0.06). The Mann–Whitney *U* test confirmed significant variance differences between mild and severe fatigue groups (*p* < 0.001). Threshold analysis identified 0.7 as the optimal operating point, achieving 90.1% precision with 84.6% recall.

## 5 Discussion

### 5.1 Adaptive time window mechanism and false alarm suppression

The confidence-driven adaptive time window approach demonstrates significant advantages for accommodating inter-individual variability and scenario-specific fatigue expression patterns while maintaining computational overhead within automotive ECU constraints. The 35.2% reduction in false alarm rates directly addresses the driver acceptance challenge that has historically impeded DMS adoption (Liu et al., 2022). The additional latency introduced by the window control logic itself is less than 5 milliseconds relative to fixed-window methods, measured as the wall-clock time consumed by confidence-to-window-length mapping and buffer length update on Jetson Xavier NX. This overhead is reported separately from the MC-Dropout cost, which is already incorporated in the overall 42 ± 5 ms per-frame inference latency reported in Section 3.4. The adaptive mechanism therefore preserves real-time processing capability essential for L2-3 takeover scenarios (Wei et al., 2023). This result validates the core design hypothesis: that confidence-driven temporal modulation achieves a superior accuracy–responsiveness trade-off compared to static window configurations, particularly under the heterogeneous fatigue manifestation patterns characteristic of naturalistic driving. Compute-matched comparisons in Section 4.3 further confirm that approximately 60–70% of the false alarm reduction originates from the confidence-driven temporal adaptation itself, with the remaining portion attributable to the ensemble effect of MC-Dropout and multi-window averaging.

Several implementation limitations warrant consideration. Nighttime highway driving with intermittent lighting or rapid tunnel transitions may generate confidence reductions that trigger unnecessary window extension despite discernible fatigue indicators (Lv and Pei, 2025). The complementary risk—that contracted windows under high confidence might miss rapid-onset fatigue events—was empirically examined in Section 4.3 and found not to elevate false negative rates relative to fixed-window baselines, although larger-scale validation on naturalistic L2-3 data remains necessary to confirm this finding. The current validation employs traditional active-driving datasets, reflecting the field-wide scarcity of large-scale L2-3 conditional automation behavioral data. L2-3 driving differs fundamentally from active control in that drivers act as passive supervisors with reduced engagement, which may alter fatigue onset latency, attention degradation profiles, and takeover-readiness dynamics. The accuracy figures reported in this study (88.6–91.8% within-dataset) therefore characterize performance on conventional driving data and should not be interpreted as direct L2-3 deployment estimates; the adaptive temporal mechanism, cross-dataset robustness, and confidence calibration framework provide a methodologically validated foundation expected to transfer to L2-3 environments, but quantitative L2-3 performance must be established once appropriate naturalistic conditional automation datasets become available. The 19.2-s mean window duration under low-confidence conditions may introduce decision latency problematic in critical takeover situations requiring rapid state transitions; future work should investigate hybrid strategies that impose upper bounds on window duration when takeover-critical contextual signals are detected.

### 5.2 Cross-dataset generalization challenges

The 10–15% cross-dataset performance degradation reflects domain shift challenges inherent to driver state estimation and is consistent with reported values in the literature, where accuracy losses of 10–25% are commonly observed across dataset boundaries (Du et al., 2020; Perkins et al., 2023). The largest degradation (13.6%) occurred in NTHU-DDD to YawDD transfer, attributable to fundamental differences in fatigue induction protocols, camera configurations, and environmental conditions. Lighting condition mismatch was identified as the dominant degradation factor, with nighttime-trained models showing reduced sensitivity under daytime conditions despite augmentation strategies (Perkins et al., 2023). Demographic differences in age, ethnicity-related facial structure, and eyewear presence contributed smaller but measurable performance reductions (Ahmed et al., 2021). Future work should prioritize the development of L2-3-specific fatigue benchmarks incorporating realistic conditional automation scenarios, standardized fatigue labeling protocols, and diverse environmental conditions to enable more rigorous generalization evaluation.

### 5.3 Implications for neurorobotics and embedded deployment

The MobileNetV3-CBAM-BiLSTM architecture demonstrates that high-accuracy fatigue detection is achievable within the computational envelope of automotive-grade embedded platforms. The 2.78 million parameter count and 328 million FLOPs represent a substantial reduction compared to prior deep architectures while delivering competitive detection performance, and the 38–45 FPS inference rate on Jetson Xavier NX confirms deployment feasibility without specialized neural processing units.

Deployment on lower-tier automotive-grade processors such as NXP S32V or Renesas R-Car V3H would require additional optimization. INT8 post-training quantization is expected to reduce model size by approximately 4× and inference latency by 2–3× with less than 1 percentage point accuracy loss, based on prior reports for MobileNetV3-family backbones, keeping the architecture within real-time bounds. Direct empirical validation on production-grade automotive chips is left to future work.

The confidence-driven feedback loop embodies a neurorobotic principle—adaptive sensory processing governed by system uncertainty—analogous to biological attentional mechanisms that modulate temporal integration windows based on signal reliability. This principle extends beyond driver monitoring to broader vehicular perception applications where reliable real-time inference under variable conditions is required, including pedestrian intention estimation and occluded object detection.

Future extensions include multimodal sensor fusion incorporating physiological signals such as electrocardiography (ECG) and electroencephalography (EEG) and vehicle behavior data (steering micro-corrections, lane deviation) for fatigue cues less susceptible to visual occlusion and illumination degradation (Gawlikowski et al., 2023; He et al., 2025). Personalization approaches establishing individual driver baselines could further reduce false alarms by accounting for idiosyncratic fatigue expression patterns and normal behavioral variability (Fan et al., 2022). Explainability enhancements through attention visualization would improve driver trust and facilitate regulatory acceptance of DMS systems in conditionally automated vehicles (Hassan et al., 2025). Transformer architectures with linear attention approximations merit investigation for extended temporal dependency modeling beyond the BiLSTM’s fixed hidden state capacity (Yang et al., 2024), while advanced UQ methods such as deep ensembles or evidential neural networks could improve calibration stability under severe distribution shift (Ren et al., 2024).

## 6 Conclusion

This paper presented a confidence-driven adaptive time window system for real-time driver fatigue detection in Level 2–3 autonomous vehicles, implemented as a deployable neuro-computational pipeline on automotive embedded hardware. The system achieved 88.6–91.8% accuracy with 0.92–0.95 AUC across four heterogeneous traditional driving datasets and demonstrated 38–45 FPS on NVIDIA Jetson Xavier NX, confirming practical deployment feasibility on automotive embedded hardware. Although the framework is designed for L2-3 conditional automation, the reported accuracy figures reflect performance on conventional driving data, and direct L2-3 deployment estimates await validation on emerging L2-3-specific datasets. The adaptive time window mechanism reduced false alarm rates by 35.2% relative to fixed-window baselines through confidence-driven temporal modulation, directly addressing a critical barrier to DMS acceptance in conditionally automated vehicles.

Cross-dataset validation yielded 75–83% accuracy with 11–14% degradation, quantifying domain shift challenges and establishing a baseline for future generalization research. Confidence calibration produced an ECE of 0.078, with high-confidence predictions (>0.9) achieving 95.6% accuracy, validating the reliability of the uncertainty quantification mechanism for adaptive warning generation.

The confidence-driven adaptive mechanism represents a practical and deployable solution for driver state monitoring in L2-3 conditional automation, providing a principled neurorobotic framework for balancing detection responsiveness with false alarm suppression under real-world variability. Future research directions include multimodal sensor fusion, personalized driver modeling, and development of large-scale L2-3 autonomous driving behavioral datasets to support continued advancement in this safety-critical application domain.

## Data Availability

The raw data supporting the conclusions of this article will be made available by the authors, without undue reservation.
